# The Ethics of Delusional Belief

**DOI:** 10.1007/s10670-015-9739-9

**Published:** 2015-06-13

**Authors:** Lisa Bortolotti, Kengo Miyazono

**Affiliations:** 1 Philosophy Department, University of Birmingham, Birmingham, UK; 2 Philosophy Department, Keio University, Tokyo, Japan

## Abstract

In this paper we address the ethics of adopting delusional beliefs and we apply consequentialist and deontological considerations to the epistemic evaluation of delusions. Delusions are characterised by their epistemic shortcomings and they are often defined as false and irrational beliefs. Despite this, when agents are overwhelmed by negative emotions due to the effects of trauma or previous adversities, or when they are subject to anxiety and stress as a result of hypersalient experience, the adoption of a delusional belief can prevent a serious epistemic harm from occurring. For instance, delusions can allow agents to remain in touch with their environment overcoming the disruptive effect of negative emotions and anxiety. Moreover, agents are not blameworthy for adopting their delusions if their ability to believe otherwise is compromised. There is evidence suggesting that no evidence-related action that would counterfactually lead them to believe otherwise is typically available to them. The lack of ability to believe otherwise, together with some other conditions, implies that the agents are not blameworthy for their delusions. The examination of the epistemic status of delusions prompts us to (1) acknowledge the complexity and contextual nature of epistemic evaluation, (2) establish connections between consequentialist and deontological frameworks in epistemology, and (3) introduce the notion of epistemic innocence into the vocabulary of epistemic evaluation.

## 1 Introduction

In “The Ethics of Belief”, Clifford defends the view that there are norms governing beliefs (*doxastic* norms), and argues that one such norm is that beliefs should be formed on the basis of sufficient evidence (Clifford 2001). In recent years, epistemologists have engaged in a lively debate on doxastic normativity and epistemic evaluation, asking whether doxastic normativity is distinctly epistemic and whether we should adopt a consequentialist or a deontological approach to epistemic evaluation. In this paper, we shall not attempt to answer such questions, but rather suggest that a closer look at the epistemic status of *delusional* beliefs can shed light on several key aspects of doxastic normativity.

In this paper, we adopt a doxastic account of delusions, and presuppose that, at least in some of the relevant circumstances, delusions are belief states. This is a widely shared view of delusions in the psychological and psychiatric literature, and it has been convincingly defended in the philosophical literature as well (e.g., Bayne and Pacherie 2005; Bortolotti 2009). That said, in philosophy there is growing scepticism about the doxastic nature of delusions (e.g., Schwitzgebel 2012; Gerrans 2014). Despite assuming doxasticism about delusions for the purposes of this paper, we believe that our argument and our conclusions will also be of interest to anti-doxasticists. In their accounts, most anti-doxasticists either concede that some delusions can be beliefs, or acknowledge that the phenomenon of delusions involves or gives rise to beliefs. For these anti-doxasticists it will be acceptable to regard some delusions or some aspects of delusions as subject to epistemic evaluation.

Now, if delusions are beliefs at all, they are beliefs that exhibit significant epistemic shortcomings. In the light of this, one may be tempted to dismiss delusions as epistemically faulty and to regard agents who adopt delusions as responsible for failing to fulfil their epistemic obligations. But, as counterintuitive as this may sound, there are reasons to believe that agents may even benefit, both pragmatically and epistemically, from having delusional beliefs and that they are not epistemically blameworthy for having such beliefs.

In consequentialist approaches to epistemic evaluation, the focus is on the consequences of an agent adopting certain beliefs or following certain rules for the adoption of beliefs. The overarching requirement is that epistemic value should be maximised when beliefs are adopted. Consequentialism takes different forms depending on how epistemic value is cashed out. For instance, veritists argue that the goal of epistemic evaluation should be the maximisation of the ratio of true to false beliefs (e.g., Goldman 1986), whereas cognitive decision theorists typically argue that the goal should be the maximisation of expected cognitive utility (e.g., Percival 2002). For virtue epistemologists, the goal is the promotion of those traits and virtues that make one a responsible epistemic agent (e.g., Greco 2012).

In deontological approaches to epistemic evaluation, the focus is on whether agents comply with the duties that apply to the adoption of beliefs (we call these *doxastic* duties). One example of a doxastic duty is the duty to adopt a belief only when sufficient evidence for it is available (e.g., Booth 2012). If agents do not comply with their doxastic duties when they can do so, then they can be blamed for having the beliefs they have.

Let us consider two cases from the literature:

**Alice the Scientist**
Alice is a scientist whose research will be funded by an organisation only if the review board of this organisation thinks that she genuinely believes in the existence of God. Alice forms the belief that God exists, because this enables her to pursue her research project and acquire new true beliefs that she would not be able to acquire otherwise.^1^

**Craig the Creationist**
Craig is told by someone he considers to be both reliable and trustworthy that there are no credible books disproving creationism. Although the question whether creationism is true is important to him, Craig does not investigate further, and thus does not acquire the evidence that might lead him to abandon his belief in creationism.^2^

In the literature on doxastic normativity, it is common to focus on the conditions for a belief to be *justified*. So, for an epistemic consequentialist committed to the view that a belief is justified when it allows the agent to achieve a legitimate epistemic goal, such as the maximisation of true beliefs, then Alice’s belief that God exists would be justified if the belief did lead her to acquire more true beliefs than false beliefs. Similarly, if an epistemic deontologist were committed to the view that a belief is justified when the person adopting that belief fulfilled her basic doxastic duties, then Craig’s belief in creationism would be justified if his evidence against creationism was limited and unconvincing or discredited by sources he had reasons to trust.

Both claims about epistemic justification seem unattractive, but there are other lessons that can be learned by exploring the case of Alice and that of Craig that are relevant to the ethics of adopting beliefs. We suggest that epistemic evaluation has a broader scope than the investigation of the conditions for justified belief. Considerations about *epistemic value* and about *epistemic responsibility and blameworthiness* are important to agents’ practices and their mutual interactions. Alice did not adopt the belief in the existence of God for purely epistemic reasons (e.g., as a result of obtaining convincing evidence for it), but her belief has some epistemic benefits nonetheless. It brings about a situation in which new true beliefs are likely to be acquired. If Craig does not have trustworthy friends telling him about credible books disproving creationism, he may not be in a position to realise that alternative hypotheses to creationism are better supported by evidence.

In this paper we apply some of the considerations above to the case of delusions. In exploring the ethics of delusion we want to consider whether adopting a delusional belief carries any epistemic benefit (Sect. 1), and whether agents are responsible and should be blamed for adopting delusions (Sect. 2).^3^ We shall approach the first question from the standpoint of epistemic consequentialism, and the second question from the standpoint of epistemic deontology. We shall argue that, at the time when the delusion is adopted, (1) the delusion may prevent serious epistemic harm from occurring by enabling the agent to overcome overwhelming negative emotions or anxiety, and (2) the agent is not epistemically blameworthy for adopting the delusion if her capacity to believe otherwise is compromised. Finally, in considering the potential implications of our view, we rely on an analogy with the legal notion of innocence defence to describe delusions as being epistemically non-wrongful, and agents with delusions as epistemically innocent.

## 2 Do Delusions have Any Positive Epistemic Consequences?

Clinical delusions are symptoms of schizophrenia, dementia, and other psychiatric disorders. Delusions are often defined in the psychological and psychiatric literature as beliefs characterised by their numerous epistemic shortcomings (e.g., APA 2013). Delusions are typically implausible and resistant to evidence, they may be badly integrated in a belief system, and they may lead to the acquisition of other false beliefs. Agents adopt delusional hypotheses despite often realising that such hypotheses are implausible, and maintain delusions in the face of both authoritative challenges and counterevidence. Can beliefs with such serious epistemic short-comings have any redeeming features?

Epistemic consequentialism seems to be the natural standpoint from which to consider the question whether delusions have positive epistemic consequences. That is because consequentialism is committed to the view that, in determining the epistemic status of a belief, we have to take into account the *total epistemic value of having the belief* and compare that to the total epistemic value of not having the belief (where this may include the value of having another belief or of suspending judgement). In this framework, even beliefs with epistemic costs can contribute to the pursuit of epistemic goals. We shall argue that delusions are a good example of epistemically costly beliefs that can also have epistemic benefits, because in some contexts, when they are adopted, they prevent serious epistemic harm from occurring. In particular, they are a means to avoiding the break-down of epistemic functionality at a critical time, when the agent is overwhelmed by negative emotions or hypersalient experience.

Our proposal is to take into account the context in which delusions are adopted. In particular, we shall look at two cases: (a) the case of *motivated delusions*^4^, that is, delusions whose formation may be affected by motivational factors, and that have been construed as playing a defensive function (McKay and Kinsbourne 2010); and (b) the case of *elaborated and systematised* delusions in schizophrenia. What psychological benefits delusions have may differ depending on some of their relevant surface features, so it is useful to group delusions according to such features. However, we acknowledge that the proposed categories of motivated delusions and elaborated and systematised delusions do not neatly map onto distinct aetiologies and are neither exclusive nor exhaustive. Some delusions are neither elaborated nor motivated (i.e., so-called “deficit” delusions such as the Capgras delusion), and delusions in schizophrenia can be elaborated, systematised and, to some extent, motivated (e.g., persecutory and grandiose delusions).

### 2.1 The Case of Motivated Delusions

Example of delusions that have been construed as playing a defensive function are the Reverse Othello syndrome and anosognosia. Such delusions have been found to have epistemic benefits that are mediated by their positive effects on wellbeing (Bortolotti 2014).

In a case of Reverse Othello syndrome (Butler 2000), a musician (BX) became quadriplegic as a result of a car accident and developed the delusion that he was still in a happy relationship with his previous romantic partner. The delusion seemed to protect BX from the undesirable truth that his romantic partner had left him while he was coping with the consequences of permanent disability. No other delusions or psychotic symptoms were observed (Butler 2000, p. 90). Butler argues that the delusion was a psychological defence against depression and this contributed to the fixity and elaboration of BX’s delusional system. The delusion kept BX’s depression at bay at a time that was critical. Acknowledging the end of his romantic relationship might have been disastrous when he was coping with the realisation of his new disability and its effects on his life.

In anosognosia the agent refuses to acknowledge a serious impairment as a result of trauma or illness, and often also fails to recognise its implications. Ramachandran (1996) advances the hypothesis that the behaviours that give rise to delusions in this context are an exaggeration of normal defence mechanisms that have an adaptive function. Denying change can sometimes be instrumental to preserving a coherent system of beliefs and behaving in a stable and predictable manner (Ramachandran 1996). The psychological advantages are not necessarily cashed out in terms of the preservation of the concept of the self *as healthy*, but in terms of the preservation of the concept of the present self *as coherent with* that of the past self. Fotopoulou has observed this phenomenon in people who do not update personal information because they “need to highlight their continuity and coherence with their past selves and may not be able to understand or deal with the loss of their previous family and social role.” (Fotopoulou 2008, p. 560).

Aimola Davies et al. (2009) report that there are both positive and negative effects of anosognosia on wellbeing, and suggest that there could be a role for motivational factors in the explanation of anosognosia. Anosognosia has negative effects in that people who do not acknowledge their illness or impairment may be slow in seeking treatment and less motivated to engage in rehabilitation. But anosognosia is associated with reduced anxiety. Its effects include: “protection from negative emotional states, reduced medical complications, and lower levels of anxiety and depression” (Aimola Davies et al. 2009, p. 200). Indeed, self-report of symptoms post-injury correlates with higher emotional distress (Gasquoine 2015).

According to the “shear-pin” account discussed by McKay and Dennett (2009), some false beliefs count as psychologically adaptive if they help manage negative emotions and avoid low self-esteem and depression. McKay and Dennett suggest that, in situations of extreme stress, motivational influences are allowed to intervene in the process of belief evaluation, causing a *breakage*. The breakage seems to be bad news from an epistemic point of view, as the result is that agents come to believe what they desire to be true and not what they have evidence for. However, the breakage is not an evolutionary “mistake”, it is designed to avoid breakages that would have worse consequences for the agent’s self-esteem and wellbeing.

Could motivated delusions be *adaptive misbeliefs*? The hypothesis is that the mechanism that inhibits motivational influences on belief evaluation becomes compromised, and thus motivated delusions emerge, making negative emotions easier to manage and depression less likely to ensue. In their paper McKay and Dennett consider the possibility that some delusions count as adaptive misbeliefs. They argue that, although there is an adaptive mechanism allowing desires to influence belief formation in situations of extreme stress, the extent to which desires influence belief formation in the case of delusions is pathological. Motivated delusions are the result of the *maladaptive* version of a psychologically adaptive mechanism.

As described by McKay and Dennett, the situation in which adaptive misbeliefs emerge is already seriously compromised. The premise is that the agent is experiencing high levels of distress, and can come to serious harm unless her negative emotions are managed. Our reading of the situation is that the adaptive misbelief is equivalent to an emergency response. Indeed, McKay and Dennett talk about the “extraordinary circumstances” in which motivational influences on belief are not just tolerated but desirable. By believing in a more positive version of reality (e.g., “I am now severely disabled, but my girlfriend is still by my side”; “I’m still as self-sufficient as I was before the accident”) than the one they have evidence for, agents manage negative feelings that could become overwhelming, avoid depression, preserve self-esteem, and overcome anxiety and stress.

McKay and Dennett focus on the effects of adaptive misbeliefs on *wellbeing* and their argument is for the *psychological* adaptiveness of delusions in some contexts. The point of allowing motivational factors to influence belief evaluation is to make the agent *feel better* about herself and her situation. But our suggestion is that the adoption of the delusion can have some *epistemic* benefits that are mediated by the psychological ones. In the given circumstances, the delusional belief is likely to have positive effects on the agent’s capacity to function *epistemically* (what we call “epistemic functionality”).

The following is an empirically plausible account of the form that the epistemic benefits can take. Although negative emotions, stress, and anxiety are a natural and often an adaptive response to traumatic and threatening situations, when they become overwhelming they are correlated with difficulties in directed thinking and recall, irritability, and emotional disturbances. More important to us, they have been robustly linked to poor short-term memory, impaired concentration, and reduced attention (see Eysenck 1992 for a classic overview, and Forster et al. 2015 for a recent study of how anxiety affects sustained attention tasks). This finding applies cross-diagnostically and there are several theories attempting to shed light on the underlying mechanisms responsible for it.

The difficulties in cognitive processing have a negative impact on both agents’ capacity to acquire new information and their socialisation, making interaction with other people less frequent and less conducive to the exchange of relevant information and to feedback on existing beliefs. Thus, due to reduced socialisation and limited engagement with the environment, the capacity to update existing beliefs and acquire new ones (which is key to epistemic functionality) is compromised. By relieving the stress caused by trauma or other adversities, the adoption of a motivated delusion allows the agent to engage with her surrounding physical and social environment in a way that is more likely to lead to epistemic achievements than the paralysing state of depression that is likely to take over otherwise.

As we mentioned earlier, our interest here is not in whether delusions are justified. We acknowledge that their potential epistemic benefits are accompanied by significant epistemic shortcomings. Because of that, the claims that the adoption of a delusion leads to the acquisition of more true beliefs than false beliefs and that delusions are epistemically beneficial overall do not seem promising. Rather, we suggest that in some circumstances the adoption of a delusion enables the agent to continue acquiring beliefs, whether true or false, and engage in other relevant practices such as giving and receiving feedback, and exchanging information. Such practices would be seriously disrupted by the epistemic break-down that overwhelmingly negative emotions, anxiety, and stress can engender after trauma. In the case of motivated delusions, the epistemic benefits we have described are mediated by the psychological adaptiveness of the delusions.

### 2.2 The Case of Elaborated and Systematised Delusions in Schizophrenia

According to one popular account of the formation of schizophrenic delusions, in the prodromal phase of psychosis, people are bombarded with stimuli presented to them as inexplicably salient (Jaspers 1963; Kapur 2003).

This general delusional atmosphere with all its vagueness of content must be unbearable. Patients obviously suffer terribly under it and to reach some definite idea at last is like being relieved of some enormous burden. […] The achievement brings strength and comfort. […] No dread is worse than that of danger unknown (Jaspers 1963, p. 98).

People do not know how to interpret the hypersalient stimuli and become anxious. The world becomes difficult to understand and predict. Anomalous experiences create puzzlement, and anxiety. The agent is constantly expecting something important to happen, until the delusional hypothesis is endorsed. With the adoption of the delusion, uncertainty is overcome and puzzling experiences are made sense of. In this context, the adoption of delusions that can become elaborated and systematised is described as both psychologically and biologically adaptive (Roberts 1992; Mishara and Corlett 2009) and is found to have some epistemic benefits too (Bortolotti, forthcoming).

Glenn Roberts argues that delusion formation is adaptive in that it allows agents to attribute meaning to their experience and overcome anxiety. He finds that patients with systematised delusions score higher than patients in remission, rehabilitation nurses, and Anglican ordinands in the purpose in life test and the life regard index (Roberts 1991). From these striking results, Roberts concludes that “elaborate delusional systems may, in part, be perpetuated and mediated by the associated psychological benefits” (Roberts 1992, p. 305).

Roberts’ findings seem consistent with more recent studies, according to which delusions confer meaning to otherwise deeply puzzling and inexplicable experiences and help enhance what has been called an overall “sense of coherence”. The sense of coherence is defined as “a global orientation that expresses the extent to which one has a pervasive, enduring though dynamic, feeling of confidence that (1) the stimuli deriving from one’s internal and external environments are structured, predictable, and explicable; (2) the resources are available to one to meet the demands posed by these stimuli; and (3) these demands are challenges, worthy of investment and engagement” (Antonovsky 1987, p. 91).

Bergstein and colleagues (2008) find that the sense of coherence is not reduced in people “in an acute delusional state”. The sense that their lives are meaningful might even be enhanced with respect to the non-clinical population, especially when the delusional system is elaborated. Sense of coherence and meaningfulness are found to correlate with wellbeing. In the transition from the acute state to remission, when conviction in the delusions fades and the new explanation for the delusional experiences involves insight into psychosis, sense of coherence and meaningfulness are reduced, and levels of wellbeing are also found to drop.

Do meaningfulness and sense of coherence translate into epistemic benefits? A first hypothesis about the epistemic benefits of elaborated and systematised delusions emerges from the claim that their formation provides relief from stress and anxiety, as in the case of motivated delusions. Here the initial anxiety would not be caused by the effects of trauma or other adversities, but by the puzzling nature of hypersalient experience.

First, endogenous psychosis evolves slowly (not overnight). For many patients it evolves through a series of stages: a stage of heightened awareness and emotionality combined with a sense of anxiety and impasse, a drive to “make sense” of the situation, and then usually relief and a “new awareness” as the delusion crystallizes and hallucinations emerge (Kapur 2003, p. 15).

The suggestion seems to be that, unless the inexplicability of salient events which is characteristic of delusional mood is resolved, great anxiety and negative emotions can become overwhelming, with adverse effects for wellbeing (Bergstein et al. 2008; Roberts 1992). As we saw, mounting anxiety and an inability to manage negative emotions can also have negative effects for epistemic functionality. In particular, lack of concentration and attention are the most frequently reported symptoms in the prodromal phase of psychosis (e.g., Yung and McGorry 1996).

A second hypothesis about the epistemic benefits of systematised delusions is that their formation engenders an attitude towards experience which promotes an active engagement with the environment and, arguably, the acquisition of new information. The agent who has formed a delusional hypothesis no longer finds her experience puzzling, but feels that it is in her power to understand it and that it is important to come to such an understanding. The sense of coherence measured by Bergstein and colleagues seems to include intellectual curiosity and a general sense of self-efficacy and purpose that would not be present in the state of passive, anxious uncertainty that characterises the agent’s experience prior to the formation of the delusion. An enhanced sense of coherence allows people to view their own experiences as interesting and worth investigating.

A third hypothesis about the epistemic benefits of systematised delusions is that delusions enable the agent “to remain in vital connection with his/her environment” (Mishara and Corlett 2009, p. 531). This is due to the effects of delusion formation on learning. Mishara and Corlett describe the process by which delusions are formed and consolidated. According to them, there is often a long period of great anxiety during which the agent is constantly expecting something important to happen. During this period, the automated and habitual processes by which we learn are disrupted due to an incorrect signalling of prediction errors, and conscious and controlled processes take over.

Attention is drawn toward irrelevant stimuli, thoughts, and associative connections which are distressing and unpredictable (Kapur 2003; McGhie and Chapman 1961; Uhlhaas and Mishara 2007). This reflects an impairment in the brain’s predictive learning mechanisms, such that unexpected events, prediction errors, are registered inappropriately (Corlett et al. 2007). (Mishara and Corlett 2009, p. 531)

Following Conrad (1958), who described the formation of delusions as a revelation, Mishara and Corlett argue that when the delusion is formed it puts an end to the overwhelming anxiety. The sense of unpredictability caused by the inaccurate coding of a prediction error stops. Attention is drawn away from the stimuli previously experienced as inexplicable and distressing, because a suitable explanation has been found for the unpredictable associations. Automated and habitual processes underlying learning resume their normal function. The delusion is stamped into memory, advocated, and reinforced every time a new prediction error is registered. The shift back to the habitual and the automated processing of learning can enhance the capacity to respond to cues in the environment. The delusion plays a dominant role in providing explanations for the phenomena previously found to be puzzling and anomalous: “the delusions […] involve a ‘reorganization’ of the patient’s experience to maintain behavioral interaction with the environment despite the underlying disruption to perceptual binding processes” (Mishara and Corlett 2009, p. 531).

Mishara and Corlett (2009) are interested in the way delusion formation in the context of schizophrenia can preserve and even enhance learning and memory. They argue that the attention and control dedicated to the unpredictable hypersalient events detract from the agent’s capacity to learn and remember. Prior to the formation of the delusion, the uncertainty caused by the unexpected associations causes conscious and controlled processes responsible for learning to focus on the stimuli that seem perplexing or threatening at the expense of the other stimuli that end up being neglected. When the delusion is formed, attention is released and habitual and automated processing resume: “the delusion disables flexible, controlled conscious processing from continuing to monitor the mounting distress of the wanton prediction error during delusional mood and thus deters cascading toxicity” (Mishara and Corlett 2009, p. 531). The adoption of the delusional belief “frees up” the agent’s cognitive resources.

### 2.3 Qualifications and Interim Conclusions

So far we have reviewed some arguments for the view that adopting delusional beliefs can have some epistemic benefits. The claim needs to be qualified, though.

Relief from stress and anxiety may be observed at the stage of *adoption* of the delusion, both in the context of motivated delusions and delusions within schizophrenia, but in the latter case the *maintenance* of the delusion often increases rather than reduces anxiety. Agents may no longer feel anxious and distressed about the consequences of their puzzling experience of hypersalience (“How should I interpret this?”), but new anxieties arise due to the often disturbing content of the delusion and the social isolation that ensues from reporting the delusion and being met with incredulity (Broome et al. 2005). Grandiose delusions are correlated with high self-esteem and low depression, but other delusions with negative content, such as delusions of persecution, are correlated with high depression and low self-esteem (e.g., Smith et al. 2006).

We saw that the formation of delusions is correlated with an increased sense of coherence and allows the agent to resume contact with her environment. We highlighted the potential benefits this may have for the agent’s attitude and the potential for acquiring new beliefs, but we should also consider that the agent with delusions will not approach the world with an open mind. New experiences will be interpreted as confirming the hypotheses that have crystallised into delusions. Every time a new salient fact is confronted, “there is a ‘monotonous’ spreading of the delusion to new experience” (Mishara and Corlett 2009, p. 531). Thus, it seems very plausible that agents with delusions are more likely to acquire new beliefs than agents puzzled by hypersalience, but some of these new beliefs are likely to be consistent with the delusional hypothesis.

Finally, one may wonder whether the features we mentioned are unique to delusions. Wouldn’t an anxiety-relief pill, a good night sleep, or a lovely meal have the same beneficial effects on epistemic functionality as the formation of the delusion (and fewer costs)? This is a good point but, from a merely consequentialist standpoint, one can just bite the bullet. If the anxiety-relief pill has the same effects as the adoption of the delusion and fewer costs, then it has a higher epistemic value. It is still significant that delusions can prevent serious epistemic harm from occurring. By paying attention to these unexpected features of delusions we contribute to providing a more balanced epistemic evaluation of epistemically costly beliefs, and we may even contribute to an explanation of delusion formation.

There is something more we can say, something that will appeal to non-consequentialists as well, about the specific contribution that delusions can make to restoring epistemic functionality. When the delusion defuses negative emotions and increases the sense of meaningfulness of one’s life, it does so via its *content*. In the case of Reverse Othello syndrome, it is because BX believes that his former partner is still faithful to him that he overcomes the negative feelings about his prospects after the car accident that caused him severe disability. In delusions of reference, it is because the agent with schizophrenia interprets hypersalient events as related to her role in some important enterprise that she can find the world around her interesting and engaging again. At least in the contexts we have discussed, the delusion acts as a belief that transforms the scenario in which the agent finds herself, and supports epistemic functionality *because of that*.

Our interim conclusion is that, in the conditions of anxiety and stress generated by the adverse effects of trauma and adversities or the salience of unpredictable stimuli, the formation of a delusion can help manage negative emotions and overcome the delusional mood characterised by hypersalience. The psychological benefits of delusions are correlated with short-term epistemic benefits that depend on the agent having her epistemic functionality compromised prior to the delusional belief being adopted. The adoption of a delusion prevents the occurrence of a disastrous epistemic break-down, but its benefits are unlikely to outlive the resolution of the break-down.

Given that the adoption of a delusion has significant epistemic shortcomings, it would be preferable to adopt a belief that supports the agent’s epistemic functionality to the same extent as the delusion, but does not have the same epistemic shortcomings. Is there an alternative to adopting a delusional belief? A thought worth exploring is that, in the extraordinary circumstances in which agents find themselves prior to developing a delusion, no other belief is available.

In Sect. 2, we shall ask whether agents who endorse delusional beliefs can believe otherwise and whether they should be considered responsible and doxastically blameworthy for their delusional beliefs.

## 3 Delusional Beliefs and Doxastic Blame

It is a common idea, inside and outside philosophy, that agents with serious mental disorders impairing agency might not be fully blameworthy for their ethically problematic actions when these are caused by their mental disorders. In a case where, for example, George with schizophrenia attacks his neighbours because of his delusion that the neighbours are trying to kill him, he might not be fully blameworthy for the attacking.^5^ This, of course, does not mean that the action is acceptable. Attacking neighbours is obviously ethically problematic. The idea is rather that George, *the agent*, is not fully blameworthy for his ethically problematic action.

In this section, we defend a similar claim with respect to whether agents are blameworthy for believing delusional hypotheses.^6^ The claim is that agents with delusions are, at least in many cases, not blameworthy for their delusional beliefs. George, for instance, is not blameworthy for the delusional belief that his neighbours are trying to kill him. This, of course, does not mean that the delusion is not epistemically faulty. In the previous section, we argued that delusions have some epistemic benefits. At the same time, though, we acknowledged that delusions have several epistemic shortcomings; most of them are false and, even when they are not, they are accidentally true at best and, thus, they do not amount to knowledge.^7^ Moreover, we noticed that delusions often do not cohere well with the agents’ other beliefs and are resistant to counterevidence. Thus, we do not wish to argue that delusions are epistemically unproblematic. Rather, we argue that agents are, at least in many cases, not blameworthy for their delusions.

In general terms, when are agents blameworthy for epistemically faulty beliefs, whether these are delusional or not? There are two extreme answers to this question. The first answer is that agents are always blameworthy for their epistemically faulty beliefs. Let us call this first view “universalism”. The second answer is that agents are never blameworthy for their epistemically faulty beliefs. Let us call this second view “nihilism”. And there is a third, modest answer according to which agents are sometimes, but not always, blameworthy for their epistemically faulty beliefs. We call this third view “restrictivism”. In the following discussion, we presuppose that restrictivism is true. The main challenge to restrictivism comes from the famous argument in Alston (1989): (1) doxastic blameworthiness implies voluntary control over beliefs; (2) agents do not have voluntary control over any of their beliefs; (3) therefore, they are never blameworthy for any of their beliefs.^8^ There are many critical responses to the argument in the literature,^9^ but there is no agreement about what exactly is wrong in the argument.

We assume restrictivism because we find it independently plausible. In addition, restrictivism makes our claim interesting. On the one hand, our claim that agents with delusions are, at least in many cases, not blameworthy for their delusional beliefs is true, but only trivially, if nihilism is true. If agents are *never* blameworthy for their epistemically faulty beliefs, then it is trivially true that agents are not blameworthy for their delusional beliefs. On the other hand, our claim is guaranteed to be false if universalism is true. If agents are *always* blameworthy for their epistemically faulty beliefs, then it is guaranteed to be false that agents are not blameworthy for their delusional beliefs. Thus, there is no room for a serious discussion about our claim within nihilism or universalism. According to restrictivism, however, there is room for a serious discussion about it. In some cases, agents are blameworthy for their epistemically faulty beliefs. In other cases, they are not. Then, there is room for investigating whether the case of an agent with delusional beliefs falls in the first or the second category.

### 3.1 On the Ability to Believe Otherwise

The crucial notion in our argument is that of the *ability to believe otherwise*, which we define in the following way:

S, with an epistemically faulty belief that p, was able to believe otherwise iff there is an evidential action A, which was available to S, such that if S had performed (not performed) A, S would have believed otherwise.

An action is an “evidential action” in case it is directly related to searching, gathering, or processing evidence.^10^ Evidential actions include: reading newspapers, paying attention to some objects, careful reasoning, and so on. An evidential action A is “available” to S in case S would have successfully performed A if S had tried to do so. Reading the newspaper in front of me is available to me because I would have successfully read the newspaper if I had tried to do so. S, with the belief that p, “believes otherwise” in case S fails to believe that p. To see how this notion actually works, think about the following two cases.

**Defence Minister**
A minister of defense has some reason to think that large-scale fraud has been perpetrated in the army. A committee is formed which studies the presumed fraud in detail and writes a long report about the situation, which they hand over to him. The minister, however, spends most of his time relaxing on the beach behind his house, enjoying the sun and eating pizzas. Consequently, he does not read a single letter of the report. As a result of that, he is ignorant of the fraud. For instance, he holds certain false beliefs about the army, beliefs for which he might nonetheless have good evidence, as long as he does not read the dossier.^11^

**Orange Juice**
Mark is writing out the shopping list for the weekly grocery shop. He goes to the fridge and sees that there is a carton of orange juice in the fridge. He forms the belief that there is orange juice in the fridge, and hence that he does not need to buy orange juice. As it turns out both of these beliefs are false. One of his housemates finishes off the orange juice, but stupidly put the empty carton back in the fridge. When Mark finds this out, he is irritated at his housemate, but he is also irritated at himself. He did not have to draw the conclusion that there was orange juice in the fridge. He was, after all, living in a student house where people do all sorts of dumb things. That his housemate might have returned an empty container to the fridge was well within the range of live possibilities.^12^

The defence minister was able to believe otherwise. Reading the report is an evidential action that was perfectly available to him. And, if he had read the report, he would have believed otherwise. Again, Mark was able to believe otherwise. Checking the container is an evidential action that was perfectly available to him. And, if he had checked the container, he would have believed otherwise.

It is tempting to think that there is an intimate connection between doxastic blameworthiness and the ability to believe otherwise. In particular, one might think that doxastic blameworthiness implies the ability to believe otherwise.

**Principle of Alternative Believability (PAB)**
If S is blameworthy for her belief that p, S was able to believe otherwise.^13^

Let us, first, look at the examples above. The defence minister seems to be clearly blameworthy for his false beliefs about the army. And, as we just noted, he was able to believe otherwise. Again, Mark seems to be blameworthy for his false belief about the orange juice, given the fact that, in his household, it was a live possibility (and he knew it) that an empty container is stupidly put back in the fridge. And, as we just noted, he was able to believe otherwise.

Now let us think about a slightly different version of *Defence Minister*, “*Defence Minister 2*”, where the defence minister tried to read the report, which is a PDF file, but failed because it was protected with a password that the minister did not know due to some administrative errors by his secretary. The minister consequently retained false beliefs about the army. Assuming that the minister had no alternative source of information about the fraud in the army, he was not able to believe otherwise. Thus, the minister does not seem to be blameworthy for his false beliefs in this case.

Let us consider, again, a slightly different version of *Orange Juice*, “*Orange Juice 2*”, where one of Mark’s housemates, who is a magician, finished off the orange juice in the container and put water into the container instead. Next, the water was transformed into something that was totally indistinguishable from orange juice by his magic. Mark checked the container and, being fooled by the magic, retained his belief that there was orange juice in the fridge. Assuming that the Mark had no alternative source of information about the orange juice, Mark was not able to believe otherwise. Mark does not seem to be blameworthy for his false belief in this case.

These cases are therefore consistent with PAB. However, PAB is controversial. A problem comes from notorious Frankfurt-style cases.^14^ Let us consider, for example, another version of *Defence Minister*, “*Defence Minister 3*”, where, just like in the original *Defence Minister*, the minister did not even try to read the report and, consequently, he retained his false beliefs about the army. Unbeknown to the minister, just like in *Defence Minister 2*, the report was protected with a password that the minister did not know and, accordingly, even if he had tried to read it, he would have still believed the same false propositions about the army. In this case, the minister seems to be blameworthy for his false beliefs. After all, he thinks and behaves in *Defence Minister 3* (i.e. spending most of his time relaxing on the beach without trying to read the report at all) in exactly the same way as in *Defence Minister* where he is clearly blameworthy. There does not seem to be any relevant difference between two cases that makes the minister blameworthy in one case but not in the other. However, assuming that the minister had no alternative source of information, he was not able to believe otherwise because of the password protection. Thus, *Defence Minister 3* looks like a counterexample to PAB.

Another possible counterexample is the case where the minister was not able to believe otherwise but the minister himself was responsible for the inability to believe otherwise. Let us consider, for example, “*Defence Minister 4*” in which, just like in *Defence Minister 2*, the minister tried to read the report, but fails because it is protected with a password that the minister does not know. As a result, he retained his false beliefs about the army. In this case, however, the password had already been given to the minister. He had just lost it because of his laziness. The password was confidential and it could not be retrieved or reissued. In this case, it looks as though the minister is blameworthy for his false beliefs about the army even though he was not able to believe otherwise. Thus, *Defence Minister 4* looks like another counterexample to PAB.^15^

One might think, however, that *Defence Minister 4* is not a counterexample to PAB because the minister was able to believe otherwise in this case. For instance, if the minister had kept the password, he would have believed otherwise. This objection fails, however, because keeping a password is not an evidential action in our sense. As we already noted, evidential actions are the ones that are *directly* related to searching, gathering, or processing evidence. Reading an email is an evidential action. Arranging one’s inbox is not. Keeping a password is, just like arranging one’s inbox, related to evidence only indirectly.

To deal with these cases, we retreat to the following claim:

**PAB2**
If S is blameworthy for her belief that p, then either (a) it is not the case that S believes that p (partly) because S was not able to believe otherwise, or (b) S is responsible for the fact that S was not able to believe otherwise.

(The right hand side of PAB2 is trivially true in the case where S is able to believe otherwise, since (a) is trivially true in such a case, and it makes the disjunction (a) or (b) trivially true as well.)

*Defence Minister 3* is not a counterexample to PAB2 because its right hand side is true in the case. Certainly, the minister was not able to believe otherwise. However, this is due to the fact that the report was locked with a password that the minister did not know, which had nothing to do with minister’s believing the false propositions. Thus, it is not the case that the minister believes the false propositions because he was not able to believe otherwise, which means that the clause (a) is satisfied in this case.

*Defence Minister 4* is not a counterexample either because, again, the right hand side is true. The minister was not able to believe otherwise and, in addition, he believed the false propositions partly because he was not able to believe otherwise due to password protection (thus, the clause (a) is not satisfied). However, he is responsible for the fact that he was not able to believe otherwise, which means that the clause (b) is satisfied in this case. He is the person who is responsible for the loss of the password, and losing the password is what makes it the case that he is not able to believe otherwise.

### 3.2 On Blameworthiness

Here is our argument for the claim that agents are, at least in many cases, not blameworthy for their delusional beliefs. Assuming that an agent S_d_ has a delusional belief that p:

1. S_d_ believes that p partly because S_d_ was not able to believe otherwise.
2. S_d_ is not responsible for the fact that S_d_ was not able to believe otherwise.
3. If S_d_ is blameworthy for her belief that p, then either (a) it is not the case that S_d_ believes that p (partly) because S_d_ was not able to believe otherwise, or (b) S_d_ is responsible for the fact that S_d_ was not able to believe otherwise.
4. Therefore, S_d_ is not blameworthy for believing p.

We maintain that this argument is applicable to at least many agents with delusions. The third premise directly comes from PAB2, which we take to be reasonable enough. At the very least, it is not refuted by Frankfurt-style cases. In the following discussion, we aim to support the first and the second premises.

To support these premises, first, we claim that many agents with delusions are not able to believe otherwise or, at the very least, that their ability to believe otherwise is significantly compromised due to some impairments and biases. Second, we argue that the inability to believe otherwise is explanatory of their believing delusional hypotheses (and, thus, the first premise is true), and also that they are not responsible for the fact that they are not able to believe otherwise (and, thus, the second premise is true).^16^

The first factor that compromises the ability to believe otherwise is the inability to regard relevant alternative hypotheses as live possibilities. If one believes a certain hypothesis, but fails to regard alternative hypotheses as live possibilities, then evidential actions are not likely to lead to believing otherwise. Suppose that the defence minister failed to find it a live possibility that large-scale fraud had been perpetrated in the army. In this case, he would not have believed otherwise even if he had read the report. He might have concluded that the report was unreliable. In the study by Freeman et al. (2004), research participants with delusions were asked to come up with alternative, non-delusional explanations of the events on the basis of which their delusions were formed and maintained. Surprisingly, three quarters of the participants failed to report any alternative hypotheses.^17^

Three quarters of the patients reported that there was no alternative explanation for their experiences. The delusion was their only explanation. This matches with clinical experience. Nevertheless, it is a striking finding. By definition a delusional belief is highly improbable. The evidence cited for a delusion is, at best, ambiguous. Yet most individuals could not report any potential alternative explanation for the ambiguous evidence however unlikely that they considered the alternative. (Freeman et al. 2004, p. 677)

The second factor is the inability to examine competing hypotheses carefully. If one believes a certain hypothesis, but is not able to examine competing hypotheses very carefully, then it is not likely that evidential actions will lead to believing otherwise. Suppose that the defence minister is not capable of examining the fraud hypothesis carefully. In this case, he might have failed to believe otherwise even if he had read the report. Huq et al. (1988) revealed that agents with delusions have the bias of coming to conclusions with less evidence in comparison to controls (the jumping-to-conclusion bias). In their experiment, research participants (delusional group, non-delusional clinical group, and non-clinical group) were requested to identify if a given jar was jar A, which contained 85 pink and 15 green beads, or jar B, which contained 15 pink and 85 green beads, on the basis of the observation of the beads drawn from the jar. They found that participants in the delusional group came to a conclusion with less information (2.22 beads drawn from the jar on average) than participants in different groups (3.6 and 4.58 beads for non-clinical group and non-delusional clinical group respectively).

Relatedly, agents with delusions have strong desire to reach conclusions. In a study by Colbert and Peters (2002), research participants (non-clinical individuals) were asked to indicate the extent to which they agreed with statements concerning *need for closure*, which is “the desire for a definite answer on some topic, any answer compared to confusion and ambiguity” (Kruglanski 1989, p. 14). They found that the tendency to delusional ideation is statistically correlated with a high need for closure (although need for closure was not correlated with the tendency to jump to conclusions). Need for closure was also found to be strong in clinical individuals with current or remitted persecutory delusions (Bentall and Swarbrick 2003).

The third factor comes from the motivational states (such as desires and emotions) that drive delusional hypotheses. If an agent’s belief is influenced by strong motivational states, then her evidential actions are not likely to lead to believing otherwise. Let us consider the scenario in *Defence Minister* again and suppose that the minister’s false beliefs about the army were motivated by his strong desire for the soundness of the army. In that case, he would not have believed otherwise even if he had read the report.^18^ The idea that some delusions are driven by motivational states has received some attention in the recent literature. As we observed in Sect. 1, so-called motivated delusions, such as Reverse Othello syndrome, erotomania and anosognosia, are likely to be driven by motivational forces.

Further, it has been suggested that persecutory delusions are the products of an externalising attribution bias, namely, the bias an agent is subject to if she attributes negative events to other agents rather than to herself. In a study by Kaney and Bentall (1989), research participants (delusional group, depressed group, and non-clinical group) were requested to answer questions about the causes of positive and negative events. They found that participants in the depressed group tended to attribute negative events to themselves, while participants in the delusional group attributed negative events to external causes and positive events to themselves. This externalising bias could be easily construed as a defence mechanism. For instance, one could argue that the bias is a means to protecting self-esteem.

[…] in normal individuals, the tendency to attribute the cause of negative events to external factors maintains self-esteem through the abrogation of responsibility. Since this tendency is significantly more marked in people with persecutory delusions, such delusions can be seen as an extreme method of maintaining self-esteem, a hypothesis which is consistent with Zigler and Glick’s suggestion that paranoia is a form of camouflaged depression. (Bentall et al. 1994, p. 334)

The existence of these three factors strongly suggests that many agents with delusions are not able to believe otherwise or, at the very least, that their ability to believe otherwise is significantly compromised.^19^

Now, it is likely that agents believe delusional hypotheses partly because of their inability to believe otherwise, given the fact that (1) they are unable to think about alternative hypotheses; (2) they are unable to examine alternative hypotheses carefully due to reasoning biases; and (3) there are motivational factors favouring their delusional hypotheses. Although the whole process of delusion formation and maintenance is not fully understood, it would be fair to expect that such impairments and biases play some role in the process. Thus, the first premise of the argument seems to be true. Moreover, agents do not seem to be responsible for the fact that they are not able to believe otherwise. After all, they are not responsible for having those impairments and biases in most cases.

## 4 Conclusions

Delusions have obvious epistemic shortcomings, and are often defined on the basis of their negative epistemic features, but a careful consideration of their epistemic status reveals that in some circumstances they prevent serious epistemic harm from occurring and that the agents adopting delusional beliefs do not fail to fulfil their doxastic duties.

In Sect. 1, we saw that delusions enable agents to manage negative feelings that could become overwhelming and provide an explanation for anomalous hyper-salient experience, putting an end to a state of anxious expectation that undermines attention and concentration. Some delusions also help agents resume learning that was previously disrupted by erroneous prediction-error signalling. In the contexts we considered, delusions support the agents’ epistemic functionality. In Sect. 2, we asked whether agents are blameworthy for their delusional beliefs. We proposed that agents are not blameworthy because, in the contexts in which delusions are formed, their ability to believe otherwise is significantly compromised due to reasoning impairments, biases, and motivational factors.

What does this tell us about the epistemic evaluation of delusional beliefs? Our discussion does not suggest that delusional beliefs are epistemically good or epistemically justified, but that they have some epistemic benefits that should be acknowledged. In the legal context, an “innocence defence” applies when an act that seems to be an offense is not regarded as wrongful. Here is an example:

Ann swings her arm and injures Ben. She faces moral condemnation and legal liability unless she can offer an explanation that absolves her of full blame. […] If Ann acknowledges that she intentionally hit Ben but did so to prevent him from detonating a bomb, she offers a justification. If she says that she decided to hit him because she was insane, she offers an excuse. (Greenawalt 1986, p. 89)

The first form of defence (called *justification defence*) applies to an act that is not regarded as an offence because it prevents serious harm from occurring. Self-defence is perhaps the most common example of a justification defence. The second sense of innocence (called *excuse defence*) applies to an act that is not regarded as an offence because the person performing the act did not know that it was an offence. Lack of capacity is a common example of an excuse defence.

When an innocence defence is advocated, the implication is that the person who performed the act should be acquitted. Can we invoke something like an innocence defence when considering the ethics of adopting a delusional belief? From a consequentialist standpoint, the adoption of a delusional hypothesis may help avoid bad epistemic consequences and it can support the agent’s epistemic functionality at a critical time (as we argued in Sect. 1). From a deontological point of view, impairments, biases, and motivational factors prevent agents from adopting an alternative belief to the delusional one, and from recognising the epistemic shortcomings of their delusions (as we argued in Sect. 2).

We propose that we have a case of *epistemic* innocence if, at the time of the agent adopting the delusional beliefs, the delusion prevents a serious epistemic harm from occurring, and no alternative beliefs are available to the agent. The notion of epistemic innocence that we have applied to the adoption of delusional beliefs can make a number of contributions to the general debate on the ethics of belief: it points to the fact that the scope of epistemic evaluation is wider than the enterprise of establishing whether a belief is justified; it forges connections between deontological and consequentialist frameworks; and it emphasises the need to take into account contextual factors in the practice of belief evaluation.

## Footnotes

1 This case is adapted from Berker (2013).

2 The case is adapted from Dougherty (2011).

3 Here we are not concerned with the question whether delusions are justified.

4 The phrase “motivated delusions” can be misleading, as it may suggest that the person *intentionally* adopts the delusional belief for some motive or other. In this context, as in the original context where the phrase was used, no assumption is made about the person’s intentions.

5 It is not our claim, however, that agents with schizophrenia are always blameless. See Broome et al. (2010) for a more detailed discussion.

6 In the following, the term “blameworthiness” primarily refers to epistemic blameworthiness or, more precisely, blameworthiness for beliefs with epistemic shortcomings.

7 Jaspers, for example, argues that a delusion of jealousy “does not cease to be a delusion although the spouse of the patient is in fact unfaithful - sometimes only as a result of the delusion” (Jaspers 1963, p. 106).

8 Although this argument could be used to motivate nihilism, Alston is not a nihilist. He grants that agents could be blameworthy for believing in some cases but insists that such a notion of blame does not provide an account of justification in epistemology.

9 See Peels (2013a, b)) for an overview.

10 The actions that are only indirectly related to evidence are not evidential actions. Reading an email is an evidential action, but arranging one’s inbox is not.

11 This case is discussed by Nottelman and Peels (2013a, b).

12 This case is discussed by Weatherson (2008).

13 See, e.g., Hetherington (2002) and Leon (2002).

14 See Nottelmann (2007) and Nottelmann and Peels (2013) for a discussion of Frankfurt-style cases in the context of doxastic responsibility.

15 We thank Jordi Fernández for raising this issue.

16 One might think, however, that the second premise is false in the case of delusions due to drug abuse. In such cases, agents are actually responsible for the inability to believe otherwise. We do not deny this claim. But it is possible that, in some cases of delusions due to drug abuse, agents are not responsible for the inability to believe otherwise because they are not responsible for the addictive drug consumption.

17 Strictly speaking, the evidence provided by this study is not sufficient to show that agents cannot believe otherwise at the time of adopting the delusion. It shows that, once agents have formed a delusional belief, they fail to regard alternative hypotheses as live possibilities. It is unlikely that available evidential actions after the formation of delusions would cause them to believe otherwise. But, the study does not show that agents fail to regard alternative hypotheses as live possibilities at the time of adopting a delusional belief. Available evidential actions prior to the formation of delusions could cause agents to believe otherwise. Our speculation is that, even prior to the formation of delusions, an agent typically regards the delusional hypothesis as the only plausible explanation of their experience. A first personal account of delusion formation process by Chadwick (2001) seems to support our speculation. However, more evidence is needed before a firm conclusion can be drawn. Methodologically, it is difficult to gather evidence relevant to the stage of adoption of the delusional hypothesis.

18 One might think, however, that the minister is blameworthy in this case. Our response depends on the details of the case. For instance, the minister is not blameworthy if it turns out that the belief is so strongly fixed by the minister’s desire that it is not responsive to obvious counterevidence. In such a case, the minister’s belief might be regarded as akin to a motivated delusion. Of course, it is not our view that agents are never blameworthy for false or irrational beliefs that are due to motivational factors. Our view is rather that agents are not blameworthy for false or irrational beliefs that are due to motivational factors in case the influence of the motivational factors is so strong that agents are unable to believe otherwise (according to our definition).

19 An objection is that these factors can also be found in non-delusional irrational beliefs such as racist beliefs. Does that mean, then, that people are not blameworthy for their racist beliefs? It is not obvious, however, that people with racist beliefs are not able to believe otherwise (according to our definition). This is an empirical question. It is certainly conceivable that some people with racist beliefs are not able to believe otherwise. But, still, it does not necessarily mean that such people are not blameworthy. The point of the case described in *Defence Minister 4* is that one can be blameworthy even if one is not able to believe otherwise when one is responsible for one’s inability. If it turns out that some people with racist beliefs are responsible for their inability to believe otherwise, then we might say that they are blame-worthy. And, perhaps it is not absurd to say (depending on the details though) that some people with racist beliefs are actually responsible for letting their biases, desires or inclinations dominate their belief formation and maintenance.
